# Parental influences on screen time and weight status among preschool children from Brazil: a cross-sectional study

**DOI:** 10.1186/s12966-019-0788-3

**Published:** 2019-03-12

**Authors:** Widjane Sheila Ferreira Goncalves, Rebecca Byrne, Marcelo Tavares Viana, Stewart G. Trost

**Affiliations:** 1 Centre for Children’s Health Research, Institute of Health and Biomedical Innovation, School of Exercise and Nutrition Sciences, Queensland University of Technology, Level 6, 62 Graham St, South Brisbane, QLD 4101 Australia; 2 Federal University of Pernambuco, Recife, PE Brazil

**Keywords:** Screen time, Parenting, Self-efficacy, Child, Obesity

## Abstract

**Background:**

Little is known about the influence of parental attributes and parental screen time behaviours on pre-schooler’s screen time and weight status in low-to-middle income countries. The purpose of this study was to examine the relationships between parental screen time, parental self-efficacy to limit screen time, child screen time and child BMI in preschool-aged children in Brazil.

**Methods:**

Three hundred eighteen parent-child dyads from Caruaru, Brazil completed a survey measuring sociodemographic data, weekday and weekend screen time, and parental self-efficacy for limiting screen time. Height and weight were measured and used to derive BMI and BMI percentile. Observed variable path analysis was used to evaluate the relationships between the parental and child variables.

**Results:**

Analyses were conducted for screen time on weekdays and weekend days. Parental screen time was positively associated with child screen time, either directly (weekdays = β = 0.27, *p* < 0.001, weekends = β = 0.24, *p* < 0.001) or indirectly through reduced self-efficacy to limit child screen time (weekdays = β = − 0.15, *p* = 0.004, weekends = β = − 0.16, *p* = 0.004). After controlling for household income, parental occupation, and parental BMI, greater child screen time on weekends, not weekdays, was associated with higher child BMI percentile (β = 0.15, *p* = 0.006).

**Conclusions:**

Parental screen time and self-efficacy to limit screen time are important influences on child screen time and weight status in pre-schoolers from Brazil. Reducing parental screen time and increasing parental confidence to limit screen time may be effective strategy to prevent overweight in Brazilian pre-schoolers.

## Background

Childhood obesity is a serious global public health problem [1]. In 2016, it was estimated that 44% of children from lower-middle income countries (LMIC) were overweight [2]. This represents an increase of more than 50% from 2000 [2]. Young children aged less than 5 years who are overweight or obese are at significantly increased risk of obesity in later childhood and adolescence [3, 4], with children of overweight or obese parents having a greater risk of being overweight or obese [4]. Obese children are more likely to experience significant short-term health problems, including asthma, sleep apnea, high blood pressure, musculoskeletal disorders, fatty liver disease, and insulin resistance [3, 5]. In later life, they are at greater risk of adult obesity, type 2 diabetes, cardiovascular disease, certain cancers, obstructive respiratory disease, reproductive disorders, and mental health problems [5].

Children’s screen time is an important contributing factor to the development and maintenance of overweight and obesity [6]. Excessive screen time increases the risk of childhood overweight and obesity by reducing physical activity engagement [7], increasing the consumption of unhealthy foods [8], and decreasing night time sleep duration [9]. In particular, exposure to television advertisements featuring unhealthy foods may encourage consumption of these foods and displace intake of healthy choices [10, 11]. On the basis of this evidence, the American Academy of Pediatrics [12] and public health authorities in Australia, New Zealand and Canada [13–15] recommend limiting screen time in children aged 2- to 5-years to no more than one hour a day. However, less than 12% of children in Australia and 25% of children in Canada are reported to meet screen time guidelines, with children aged 5 years watching an average of 1.2 and 1.9 h per day, respectively [16, 17]. Therefore, it is important to identify modifiable correlates of preschool children’s screen time behaviours to target in family-based obesity prevention interventions.

Consistent evidence shows that parental screen time and parental self-efficacy to limit screen time are important determinants of screen time in young children [18]. A systematic review of 30 studies identified parental screen time as the most frequently studied and consistent parental influence on screen time in children aged ≤6 years [18]. For example, in one study conducted in the United Kingdom, the risk of a child watching more than 4 h of television per day was 4 to 10 times higher if the child’s parent watched 2 to 4 h (for girls), or more than 4 h (for boys) of television per day, respectively [19]. The systematic review also found consistent evidence that parental self-efficacy to limit child screen time was inversely associated with child screen time [18]. Indeed, the significance of parent’s self-efficacy perceptions was underscored by the results of recent Australian study in which parental self-efficacy and rules to limit screen time were the only significant correlates of screen time among male and female preschool-aged children [20]. On the weight of this evidence, it seems prudent for interventions to limit screen time in young children include strategies to decrease parental screen time and increase parental self-efficacy to limit screen time. However, because previous studies were exclusively conducted in Europe, North America, and Australia [18], it is uncertain whether the findings can be generalised to families residing in LMIC’s. Currently, we are aware of no studies that have investigated the relationships between parental screen time, parental self-efficacy to limit screen time, child screen time, and child weight status in LMIC families.

To address this knowledge gap, the aim of this study was to examine the interrelationships between parental screen time, parental self-efficacy for limiting screen time, child screen time, and child body mass index (BMI) in low income Brazilian families. We hypothesized that parental screen time would significantly influence child screen time, either directly, or indirectly through its negative impact on parental self-efficacy to limit screen time. We further hypothesized that child screen time would be positively associated with child weight status.

## Methods

### Participants and setting

Seven Early Childhood Education and Care (ECEC) centers from the municipal education network of Caruaru – Pernambuco, Brazil were randomly selected and recruited into the study. All parents and their respective children aged 3 to 5 years enrolled at those centers were invited to participate in this study. Children were ineligible to participate if parents reported the child as having special needs or taking medication regularly that may influence weight. Parents who self-identified as having health problems or who were unable to have their height and weight measured were also ineligible to participate. Recruitment and data collection activities were completed between July and October 2017. To have 80% power to detect differences as small as 0.16 between the observed correlations and the null hypothesis correlation of 0.00, a sample of size of 304 parent-child dyads was required. The research was approved by the Human Research Ethics Committee of the Federal University of Pernambuco, Recife/Brazil (Opinion No. 2,145,702).

### Parent measures

One parent or caregiver from each family completed a written questionnaire as they were dropping off or picking up their child from the center. Members of the research team interviewed parents who were unable to complete the questionnaire due to low literacy levels. Where parents had multiple children enrolled in the center they were instructed to complete the questionnaire in relation to their first born.

The questionnaire included the following socio-demographic information: child’s sex, ethnicity, date of birth, attendance at ECEC (part-time vs full-time), proportion of female and male caregivers, marital status, occupation, household income, level of education, number of residents, and number of televisions at home. The majority of surveys were completed by mothers (89.6%).

Parent and child screen time was reported by parents using an instrument developed by He et al. [21]. Parents were asked to report their child’s usual time per day in screen-based activity with the following question: “How many hours does your child usually spend watching television or videos and playing computer and video games?” Weekday and weekend screen time were assessed individually. Response options were: < 1 h, 1 to 2 h, and ≥ 3 h. The child screen time items have evidence of acceptable validity (ICC’s ranged from 0.5–0.8) when compared to the activity diary method, as well as excellent test-retest reliability (ICC = 0.98) [21]. The same items were used to assess parental screen time on weekdays and weekends.

Parental self-efficacy for limiting screen time was assessed using three items from the parenting self-efficacy scale used in the Infant Feeding Activity and Nutrition Trial (InFANT Study) [22] . Parents were asked how confident they were that they would be able to do the following: 1) turn off the TV during meal times; 2) get the child to do some active play when he/she wants to watch TV; and, 3) keep the child entertained without using TV/videos/DVDs. Responses were recorded on a four point Likert scale ranging from 1= ‘not at all confident’ to 4 = ‘extremely confident’. A total score for parent self-efficacy was calculated by averaging responses to the three items, with a higher score indicating higher self-efficacy to limit screen time. The internal consistency of the scale, as measured by Cronbach’s alpha, was 0.56.

### Anthropometric measures

Height and weight were collected using standardized WHO measurement procedures [23]. Height was measured to the nearest millimeter using a portable stadiometer (Seca 213 portable stadiometer). Weight was measured to the nearest 0.050 kg using high precision digital scales (Seca 803B digital scale). During data collection, children wore standard ECEC uniforms and parents were instructed to wear light clothing, without shoes. Body mass index (BMI) was calculated according to the equation: weight (kg) / height (m)^2^ [24]. Parents were classified as overweight if their BMI was ≥25. The calculations to obtain children’s BMI percentiles were performed using WHO Anthro software, Version 3.2.2 [25], which is based on the WHO growth charts for 0–5 year olds [26]. Children were classified as overweight if their BMI was greater than or equal to the sex-and-age-specific 85th percentile.

### Statistical analysis

Path analyses using observed variables were conducted to evaluate the relationships between the parent and child variables. It was hypothesized that, after controlling for household income, parental occupation, and parental BMI; parent screen time would be inversely related to parental self-efficacy to limit screen time, with self-efficacy to limit screen time, in turn, inversely associated with child screen time. A direct positive association between parental screen time and child screen time was also tested. Child screen time and parental weight status were hypothesized to be direct influences on child BMI percentile. Prior to the analysis, data were examined for missing values. One case was removed due to having missing data for six variables. Two additional cases had responses missing for one and two self-efficacy items, respectively. For these two cases, missing values were imputed using Full Information Maximum Likelihood. Separate models were tested for weekday and weekend screen time. The analysis was conducted using maximum likelihood estimations, and model fit determined using accepted reference ranges of the normed chi-square (*x*^2^/df), comparative fit index (CFI), goodness-of-fit index (GFI), root mean square error of approximation (RMSEA) with 90% confidence interval [27]. Analyses were conducted in IBM SPSS AMOS 23.0.

## Results

Of the 675 eligible parents, 318 (47.1%) consented to participate in the study. The majority of children were male (57%), mixed-race (50%), and attended child care full time (86%), and had a mean age of 4.3 ± 0.6 years. Approximately one-third (34.4%) of children were classified as overweight or obese. Descriptive information for parents is presented in Table 1. Nearly 90% of parents participating in the study were the mothers and more than 60% identified as a single parent.

**Table 1:** Parent descriptive statistics

Variables	Parents	
Sex	n	%
Female caregiver	285	89.6
Male caregiver	33	10.4
Age (mean ± SD)	31 ± 8.7 years	
BMI*		
Non-overweight	122	39.4
Overweight/Obese	188	60.6
Marital status		
Single	198	62.5
Married	100	31.5
Separated/divorced	12	3.8
Widowed	7	2.2
Employment status		
Not employed	123	38.8
Yes – at home	73	23.0
Yes – not at home	121	38.2
Household income**		
< 1 wage	161	50.8
= 1 wage	114	36.0
> 1 wage	42	13.2
Level of education		
No study	7	2.2
Elementary school	180	56.8
High school	111	35.0
Tertiary education	19	6.0
Number of residents		
≤ 4	223	70.3
> 4	94	29.7
Number of TVs at home		
=1	213	67.2
> 1	104	32.8

*Overweight BMI > 25 kg/m^2^

**1 wage was equivalent to R$937 monthly in Brazilian Real in 2017

**Fig. 1:**
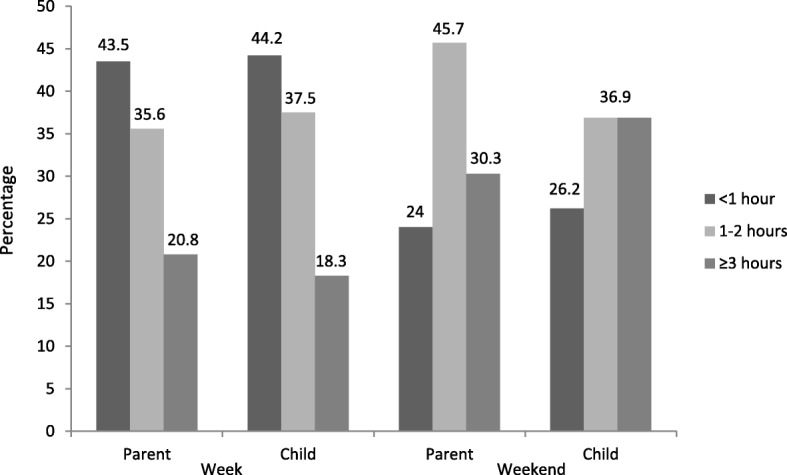
Parent and child screen time exposure on weekdays and weekends

Parent and child weekday and weekend screen time are presented in Fig. 1. The percentage of parents reporting ≥3 h of screen time was significantly higher on weekend days (30.3%) than weekdays (20.8%) (χ^2^(1, *N* = 317) = 76.3, *p* < .001). Similarly, the percentage of children with ≥3 h of screen time was significantly higher on weekend days (36.9%) than weekdays (18.3%) (χ^2^(1, *N* = 317) = 74.1, *p* < .001).

**Fig. 2:**
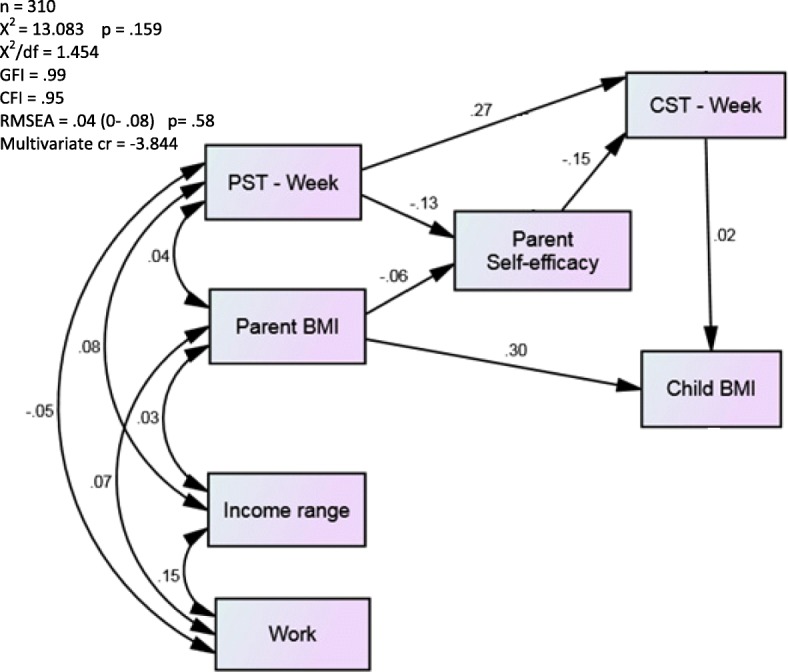
Path analysis examining the relationships between parental weekday screen time (PST-Week), parental self-efficacy for limiting screen time, child weekday screen time (CST – Week), and child BMI percentile, controlling for parent BMI, household income, and parental occupation (work). The errors terms have been removed for ease of interpreting the diagram

The path analysis for weekday screen time is shown in Fig. 2. For ease of interpretation, standardized regression weights and *p*-values are reported in the figure. The model exhibited a good fit (X^2^ = 13.083 *p* = .159; X^2^/df = 1.454; GFI = .099; CFI = .95; RMSEA = .04 (90% CI: 0–.08); Multivariate cr = − 3.844). Parent screen time was positively associated with child screen time (*B* = 0.26, 95% CI = 0.16, 0.36), and inversely related to parental self-efficacy to limit screen time (*B* = − 0.12, 95% CI = − 0.23, − 0.01). Parent self-efficacy to limit screen time was, in turn, inversely associated with child screen time (*B* = − 0.15, 95% CI = − 0.25, − 0.05). Parent BMI was positively related to child BMI percentile (*B* = 1.7, 95% CI = 1.1, 2.3); however, the relationship between child screen time and child BMI percentile was not significant (*B* = 0.64, 95% CI = − 3.6, 4.9).

**Fig. 3:**
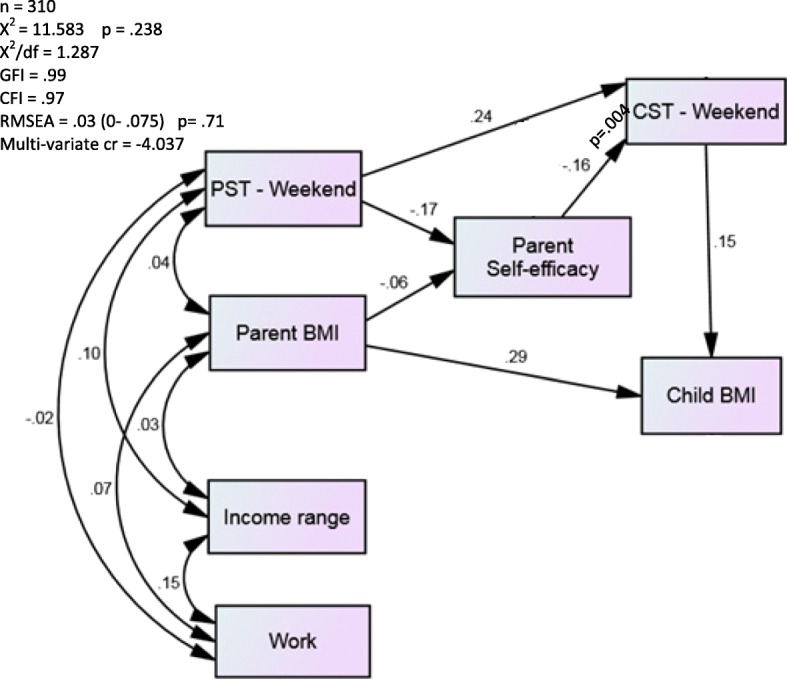
Path analysis examining the relationships between parental weekend day screen time (PST-Weekend), parental self-efficacy for limiting screen time, child weekend day screen time (CST – Weekend), and child BMI percentile, controlling for parent BMI, household income, and parental occupation (work). The errors terms have been removed for ease of interpreting the diagram

Path analysis for weekend screen time is shown in Fig. 3. For ease of interpretation, standardized regression weights and *p*-values are reported in the figure. This model also exhibited a good fit (X^2^ = 11.583 *p* = .238; X^2^/df = 1.287; GFI = .099; CFI = .97; RMSEA = .03 (90% CI: 0–.075); Multivariate cr = − 4.037). Parent screen time was positively associated with child screen time (*B* = 0.25, 95% CI = 0.14, 0.37), which in turn was inversely related to parental self-efficacy to limit screen time (*B* = − 0.17, 95% CI = − 0.29, − 0.06). Parental self-efficacy to limit screen time was, in turn, inversely related with child weekend screen time (*B* = − 0.16, 95% CI = − 0.28, − 0.05). Parental BMI (*B* = 1.6, 95% CI = 1.0, 2.1) and child weekend screen time (*B* = 5.6, 95% CI = 1.6, 9.6) were positively associated with child BMI percentile.

## Discussion

This study investigated the relationships between parental screen time, parental self-efficacy for limiting screen time, child screen time and child BMI percentile. To our knowledge, it is the first study examining these relationships in families living in under-resourced conditions of Brazil (and broadly among LMICs). In support of our hypotheses, parental screen time was positively associated with children’s screen time, either directly or indirectly through the effects of reduced self-efficacy to limit screen time. Child screen time was positively associated with child BMI percentile, but only the relationship between weekend screen time and BMI was statistically significant. Consistent with previous research, parental BMI was positively correlated with child BMI percentile [28–30]. These findings suggest that intervention programs to limit screen time in families from LMICs should focus on parental screen time and self-efficacy to limit child screen time.

Child screen time on weekends, but not weekdays was significantly and positively associated with child BMI percentile. A potential explanation for this discrepancy is the reduced amount of time available for screen time on weekdays compared to weekend days. In our study, approximately 80% of children attended ECEC centres for the full day on weekdays, where they were exposed to a structured environment with limited or no access to screens. As a result, the overall amount and between-subject variation in screen time behaviour was lower on weekdays, potentially making it more difficult to detect relationships with child weight status. This explanation is consistent with the results of a recent review examining children’s obesogenic behaviors (including screen time) on summer vacation days versus school days [31]. In that review, 155 of the 190 studies examined (81%) reported greater unfavorable obesogenic behaviors on weekend days, emphasizing that when a child is exposed to a less structured environment, children are inclined to be more sedentary. Therefore, the non-significant relationship between child screen time on weekdays and child weight status is likely a consequence of the reduced discretionary time and less opportunity for excessive screen time while attending child care.

Parental self-efficacy to limit screen time showed a significant inverse relationship with child weekday and weekend screen time. This finding is consistent with previous studies from high-income countries. A cross-sectional study of 746 children aged 0–5 years from Canada found that parental self-efficacy to limit screen time was inversely associated with child screen time [32]. Three studies conducted in Australian preschool-aged children reported parental self-efficacy to limit screen time to be inversely associated with child screen time [20, 33, 34]. Finally, a UK study of 252 preschool-aged children showed that high parental self-efficacy for limiting screen time was associated with a 77% reduction in the proportion of children watching ≥2 h of television daily [35]. Therefore, there is substantial recurrent evidence that increasing parental self-efficacy to limit screen time is a promising approach to reduce child screen time in high-income populations. This study provides new information on this topic, highlighting that early life programs to prevent excessive screen time in Brazilian families or comparable LMICs should target parents as the agent of change, and develop strategies to enhance parent’s confidence in their ability to limit their child’s screen time.

On both weekdays and weekend days, parental screen time was positively associated with child screen time. This finding is consistent with previous studies examining the relationship between parent and child screen time. A study analysing 2300 US children aged 0–8 years, found that parental screen time was strongly associated with child screen time [36]. Results from a cross-sectional study in 465 US children aged ≤5 years, reported that parental television time had a significantly stronger association with children’s television time than parental rules about television viewing or having a television at a child’s bedroom [37]. In a study of 910 Asian children aged 2 and 3 years, maternal television viewing time was one of the strongest predictors of children’s television viewing time and children’s total screen viewing time [38]. A cross-sectional study investigating the relationship between Australian and Belgian parents’ and preschooler’s television viewing reported a positive association in both samples, with a mean increase of 19.8 min and 15.0 min, respectively, on children’s screen time for each additional hour parents spend on television viewing weekly [39]. Therefore, our results, supported by other findings, indicate that even in the early years, high parental exposure to screen time may contribute to an increased risk of excessive child screen time in children residing in LMICs. This suggests that educating parents about limiting their own screen time behaviours and serving as positive role models might be an important strategy for intervention programs aiming to reduce child screen time.

This study had a number of strengths. To our knowledge, it is the first study conducted in an LMIC community to examine the relationships between screen-related parenting practices, child screen time, and child BMI. Notably, the Northeast region of Brazil has the highest proportion of adults unable to read or write (16.2%) and the lowest average monthly income (R$ 1223) compared to other regions of Brazil [40]. Second, the use of path analysis allows a comprehensive examination of multiple variables in a single analysis. The study also contributed new information about how weekday and weekend day screen time may differentially impact child BMI percentile.

There were however a number of limitations. Due to the cross-sectional study design, we cannot infer causal relationships between the parental screen time influences and the child outcome variables. Therefore, it is suggested that future research establish the temporal sequence between the parental exposures and child outcomes through longitudinal studies. Second, even though the analysis proposed in this study did not address parental limiting of screen time directly, limiting child screen time has been shown to be a consistent predictor of reduced screen time in children [18]. Third, even though the items used to quantify child screen time have established evidence of reliability and validity, the psychometric properties have not been formally assessed in Brazilian families [21]. However, in piloting, the test parents did not report any problem in understanding the content of the question, or how it should be answered. Nevertheless, future studies should assess the psychometric properties of the instrument used to measure parent and child screen time, as well as parental self-efficacy for limiting screen time in families in Brazil. Finally, only a small percentage of parents completing the survey were fathers, but previous studies have also reported low participation rates among fathers relative to mothers [39, 41–43].

## Conclusions

In summary, higher parental screen time was associated with higher child screen time, either directly or indirectly through parent’s lower self-efficacy to limit child screen time. Child screen time on the weekend was positively associated with child BMI percentile; however, the relationship between screen time on weekdays and child BMI was non-significant. The findings support the concept that interventions to decrease parent screen time and increase confidence to limit screen time may be effective in reducing screen time and promoting healthy weight in Brazilian preschool children and similar LMIC communities worldwide.

## Abbreviations

BMI: Body mass index
ECEC: Early Childhood Education and Care
LMIC: Low-middle income countries
WHO: World Health Organization
